# Climate change contributions to future atmospheric river flood damages in the western United States

**DOI:** 10.1038/s41598-022-15474-2

**Published:** 2022-08-12

**Authors:** Thomas W. Corringham, James McCarthy, Tamara Shulgina, Alexander Gershunov, Daniel R. Cayan, F. Martin Ralph

**Affiliations:** Center for Western Weather and Water Extremes (CW3E), Scripps Institution of Oceanography, University of California San Diego, La Jolla, CA USA

**Keywords:** Climate sciences, Environmental social sciences, Natural hazards

## Abstract

Atmospheric rivers (ARs) generate most of the economic losses associated with flooding in the western United States and are projected to increase in intensity with climate change. This is of concern as flood damages have been shown to increase exponentially with AR intensity. To assess how AR-related flood damages are likely to respond to climate change, we constructed county-level damage models for the western 11 conterminous states using 40 years of flood insurance data linked to characteristics of ARs at landfall. Damage functions were applied to 14 CMIP5 global climate models under the RCP4.5 “intermediate emissions” and RCP8.5 “high emissions” scenarios, under the assumption that spatial patterns of exposure, vulnerability, and flood protection remain constant at present day levels. The models predict that annual expected AR-related flood damages in the western United States could increase from $1 billion in the historical period to $2.3 billion in the 2090s under the RCP4.5 scenario or to $3.2 billion under the RCP8.5 scenario. County-level projections were developed to identify counties at greatest risk, allowing policymakers to target efforts to increase resilience to climate change.

## Introduction

### Atmospheric rivers drive flood damages in the western United States

Atmospheric rivers (ARs) are synoptic-scale features in the lower troposphere, thousands of kilometres long and hundreds of kilometres wide, that transport large quantities of water vapor from the tropics poleward (on average, more than double the mean instantaneous flow at the mouth of the Amazon River;^1^). These high flows of water vapor can cause extreme precipitation, particularly on the west coasts of major land masses as moist air rises over mountainous topography^2^. ARs were first characterized using weather model data^3^. Satellite and aircraft observations then confirmed the modelling results^4^ and documented the role of ARs in causing significant floods^5^. While ARs provide beneficial contributions to water supplies in the mid latitudes^6,7^, they also generate significant flooding. In the western United States over the past 40 years, ARs caused over $1 billion a year in average estimated flood damages, accounting for 84% of total flood damages^8^. Several billion-dollar flood events have been attributed to ARs, including the February 2017 storm that damaged the Oroville Dam in Northern California leading to the evacuation of over 180,000 people from the Feather River basin^9,10^.

With rising sea surface temperatures and increased vertically integrated horizontal vapor transport (IVT), ARs are projected to increase in intensity^11–19^, a process which appears to have already started^20^. Warming-boosted ARs encountering western topography are projected to produce more frequent and stronger precipitation extremes^18^ even while the frequency of light and moderate precipitation decreases^18,21,22^. The probability density function of daily precipitation is projected to shift in favour of extreme events due to thermodynamically increased AR intensity, while the frequency of low and medium intensity precipitation decreases^22^ due to non-AR storms^18^ being pushed poleward by the expanding subtropical belt^23^.

Flood damages associated with ARs have been found to increase exponentially with AR intensity^8^ so even a modest shift towards higher-intensity ARs could have significant impacts on flood damages. In their projections of AR-related flood damages Rhoades et al.^24^ consider the impacts of climate change under three Community Earth System Model (CESM) stabilized warming scenarios. Under a 0.85 °C warming scenario they predict that damages by the end of the century would remain in the historical $1b per year range but under a 3 °C warming scenario they predict that average damages would increase above $3b per year. Their study considers a set of outputs from a single global climate model (GCM) and models flood damages based on AR loss statistics aggregated over the entire western United States.

In this study we build county-level AR-related flood damage projections based on 16 GCMs under the RCP8.5 “high emissions” scenario using statistical models that link damages to AR landfall characteristics using historical reanalysis data. To compare expected damages under different greenhouse gas concentration trajectories, we applied the same statistical damage models to a 14-member subset of these GCMs for which sufficient AR data were available under the RCP4.5 “intermediate emissions” scenario. The use of an ensemble of GCMs allows us to quantify climate model uncertainty. County-level damage modelling allows us to generate location-specific projections which could be used to target investments in flood mitigation infrastructure at state and county levels.

While observed flood damages in the western U.S. have remained relatively constant over recent decades^25^, flood damages across the entire U.S. have increased^26^. It is important to note that these U.S.-wide increases in damages have been attributed to increased exposure and wealth, rather than climate-related changes in the frequency or intensity of the flood hazard^27–29^. In this study, we estimate the impacts of projected changes in AR frequency and intensity over the twenty-first century under the simplifying assumption that exposure and vulnerability will remain at historical levels. However, population and wealth in the western United States are projected to increase in the coming decades while vulnerability will likely decrease with continued investments in adaptation and flood prevention. These factors will alter the AR-related flood damage futures presented here.

### Damage projections over the western United States

Forty years of flood insurance data from the National Flood Insurance Program (NFIP)^8^ linked to total direct damage estimates from the National Weather Service (NWS)^27^ and an observational catalogue of landfalling AR events^30^ were used to build statistical models of county-level flood damages as functions of characteristics of ARs at landfall from Baja California to British Columbia. NFIP insured losses and total direct damage estimates by county and over the western United States were modelled as functions of landfalling total IVT by latitude, AR ranking, seasonal or month-of-year indicator variables, and amount of time since last AR (Supplementary Materials). The models were applied to past and future synthetic AR catalogues derived from CMIP5 (Coupled Model Intercomparison Project 5) global climate models under the RCP4.5 and RCP8.5 (representative concentration pathway 4.5 and 8.5 W/m^2^) climate scenarios from the year 1950 to 2100^18,31^.

AR catalogues for RCP8.5 were available for 16 GCMs. These GCMs (Table S1) were chosen because they contained sufficient information to calculate daily IVT, integrated vapor transport, a critical AR intensity metric which measures the amount and velocity of water vapor traveling through the atmosphere over a given location^18^. At the time of analysis, the RCP4.5 r1i1p1 ensemble data were available for only 14 of these GCMs, due to technical issues. Results comparing RCP4.5 to RCP8.5 are based on the 14-member subset of GCMs (Table S1). All other results are presented for RCP8.5 only, using the full set of 16 GCMs (Table S1). Using the statistical damage models based on AR characteristics, each synthetic AR in each of the AR catalogues was assigned a flood damage estimate at the county level, and across the western United States. The storm damages were then aggregated annually to form regional and county-specific time series of projected damages (Supplementary Materials).

**Figure 1:**
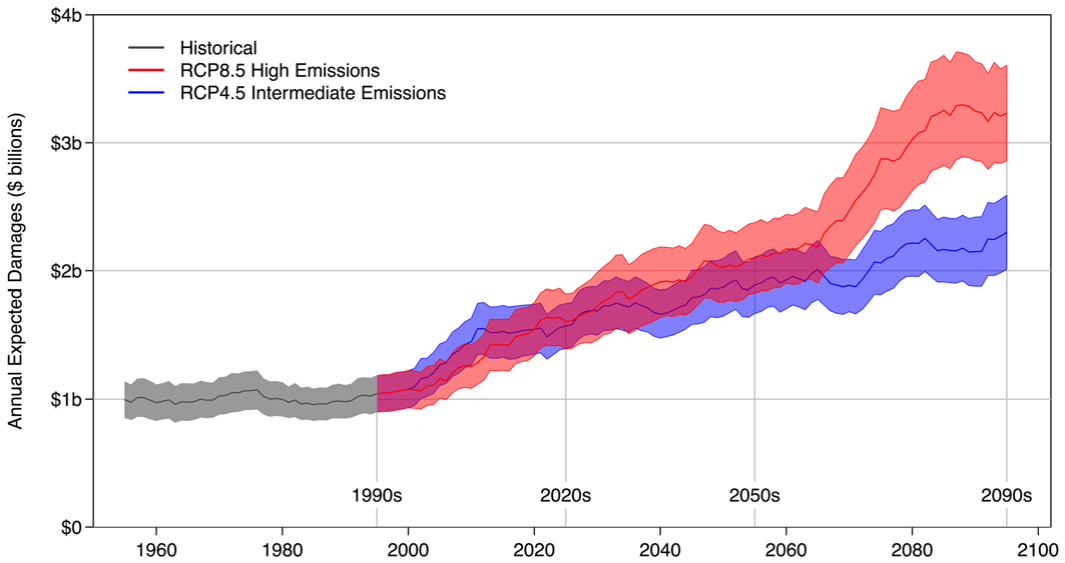
Projections of AR-related flood damages are generated using a loss model linked to 14 GCMs. Expected annual damages remain stable over the historical period and begin to diverge beyond the 2020s. Expected damages under the RCP8.5 “high emissions” scenario double by the 2050s and triple by the 2090s relative to the reference level of the 1990s. Means and 95% standard errors are taken over centred 10-year moving windows of observations from the 14-model ensembles.

In a 14-model comparison of projected damages between the RCP4.5 and RCP8.5 scenarios, expected annual damages rise from $1 billion in the 1990s to $1.6 billion in the 2020s for both climate scenarios (Fig. 1). By the 2050s, projected damages are $1.9 billion and $2.1 billion for RCP4.5 and RCP8.5, respectively. By the 2090s projected AR-related flood damages across the western United States are $2.3 billion under RCP4.5 and $3.2 billion under RCP8.5 (all damages are adjusted for inflation and reported in 2022 U.S. dollars). The RCP8.5 ensemble mean is within the 95% standard error range of the RCP4.5 models in the 2020s and 2050s but above the 95% standard error range of the RCP4.5 models in the 2090s. Under the assumptions of this modelling framework, limiting greenhouse gas concentrations to the RCP4.5 trajectory relative to the RCP8.5 trajectory would reduce projected end-of-century AR-related flood damages by 30% across the western United States saving almost $1 billion a year. Projections of NFIP insured losses normalised by dividing by annual West-aggregated NFIP coverage in force (see, e.g.,^29^) yield similar qualitative results indicating some robustness of the models to the variability in exposure over the historical period (Fig. S1).

**Figure 2:**
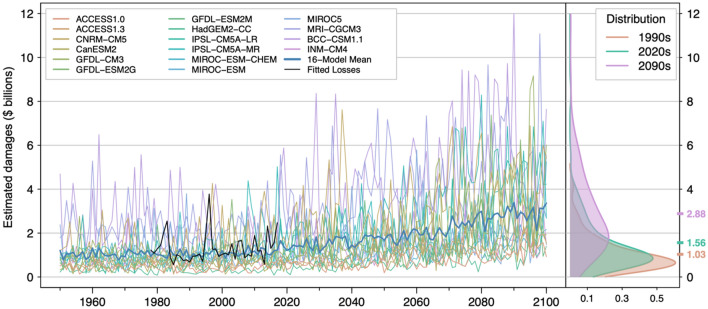
The 16 RCP8.5 climate models predict an increase in mean annual damages over the 11 western states from $1.03 billion in the 1990s to $1.56 billion in the 2020s to $2.88 billion in the 2090s. MRI-CGCM3 and BCC-CSM1.1 generate the highest damage projections; ACCESS1.0 and HadGEM2-CC generate the lowest projections. Gaussian kernel densities of damages by decade across the 16 models reveal increasing damages over time (right panel). The shift in the distribution is more apparent by the 2090s with an increasing number of model years in the upper tail of the distribution. Kolmogorov–Smirnov tests on the differences in annual damage distributions in the 2020s and 2090s relative to the 1990s yield significant *p*-values of 0.006 and less than 1 × 10^–16^, respectively.

The full set of 16 projections for the RCP8.5 scenario exhibit considerable variability (Fig. 2). The ensemble mean of the synthetic damage time series shares the same qualitative characteristics as the fitted values of the model over the observational period: annual AR flood damage estimates for the 11 western conterminous states range from $100 million to $5 billion; the distribution of damages is positively skewed with most of the total damages concentrated in a small number of highly damaging storms. In contrast to the 14-model RCP8.5 projections, the 16-model ensemble mean of annual estimated damages is $1.0 billion in the 1990s, $1.6 billion in the 2020s, and $2.9 billion in the 2090s. Both the 14- and 16-model ensembles, combined with the statistical damage model, project that expected annual flood damages due to ARs will approximately triple from the 1990s to the 2090s under the RCP8.5 scenario. This is in line with previous results by Rhoades et al.^24^.

Consistent with AR intensity projections for this region^18^, the flood damage projections are relatively stationary over the latter half of the twentieth century but begin to show an increasing trend in the first half of the twenty-first century (Fig. 2, Tables 1 and 2). Comparing the empirical distributions of annual damage estimates by decade, the model damages in the 2020s differ significantly from the damages in the 1990s, with a Kolmogorov–Smirnov test *p*-value of 0.0062. By the 2090s the decadal distribution of damages is significantly different from that of the 1990s with a *p*-value < 1 × 10^–16^. The multi-model distribution of damages exhibits greater variance by the end of the century and greater positive skewness, indicating the potential for more frequent highly damaging events. We note that the increases in multi-model ensemble mean damages from the 1990s to the 2020s are not borne out in the observational record. This may be due to the stochastic and intermittent nature of highly damaging flood events. 14- and 16-model ensemble means represent expected damages while the observational record is one realization of the set of possible damage time series over the historical period.

**Table 1:** Annual estimated damages over the western 11 states, calculated using decadal moving windows of projections from 16 global climate models, increase by 180% from the 1990s to the 2090s. Differences in distributions of model projections become statistically significant by the 2020s at *p* < 0.01.

Insured Losses and Total Damages 1950 to 2090 relative to 1990				
Decade	Insured loss ($M)	Estimated damage ($B)	Percent change	K.S. *p*-value
1950	33.3	1.00	-3.2	0.41
1960	34.5	1.04	0.2	0.78
1970	36.5	1.10	6.0	0.92
1980	35.0	1.05	1.6	0.75
1990	34.4	1.03	0	1
2000	36.5	1.10	6.0	0.46
2010	41.0	1.23	19.0	0.18
2020	47.6	1.43	38.2	0.0062
2030	52.0	1.56	51.0	0.00076
2040	56.9	1.71	65.2	1.6 × 10^–6^
2050	60.8	1.82	76.6	2.5 × 10^–9^
2060	65.7	1.97	90.8	1.2 × 10^–10^
2070	82.6	2.48	139.9	2.2 × 10^–15^
2080	92.6	2.78	168.9	1.1 × 10^–16^
2090	96.2	2.88	179.4	< 1.0 × 10^–16^

**Table 2:** Annual estimated damages over the western 11 states increase by roughly 10% per decade over the next three decades relative to the current 2020 baseline. Differences relative to current conditions become statistically significant by 2040 at *p* < 0.05 and 2045 at *p* < 0.01.

Insured Losses and Total Damages 2020 to 2050 relative to 2020				
Year	Insured loss ($M)	Estimated damage ($B)	Percent change	K.S. *p*-value
2020	44.13	1.32	0	1
2025	47.59	1.43	7.8	0.91
2030	48.55	1.46	10.0	0.57
2035	52.02	1.56	17.8	0.20
2040	54.79	1.64	24.1	0.02
2045	56.91	1.71	28.9	0.0072
2050	59.01	1.77	33.7	0.0014

**Figure 3:**
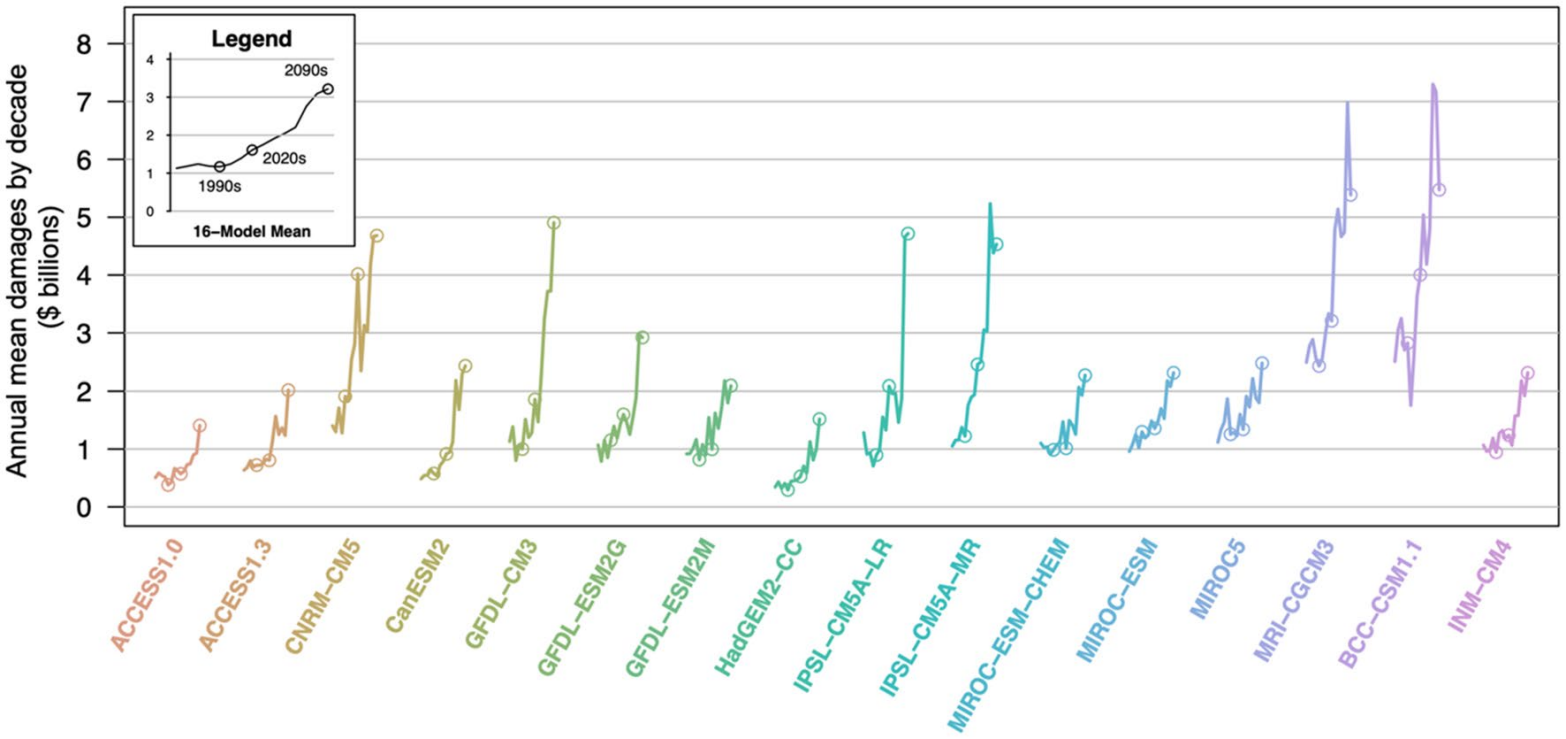
Each of the 16 models predict increases in annual mean damages by decade over the 11 western states from the 1950s to the 2090s, with the most significant increases occurring in the latter half of the twenty-first century. Six models stand out with high end-of-century predictions: CNRM-CM5, GFDL-CM3, IPSL-CM5A-LR, IPSL-CM5A-MR, MRI-CGCM3, and BCC-CSM1.1. The remainder of the models predict lower damages, with ACCESS1.0 and HadGEM2-CC at the lower end of the set. The 16-model mean (inset) shows annual damages by decade increasing monotonically over the sample period.

All 16 models project increases in AR-related flood damages by the end of the century (Fig. 3). Of the 16 synthetic AR damage time series, those based on the Japanese model MRI-CGCM3 and the Chinese model BCC-CSM1.1 project the highest damages by the 2090s, while those based on the Australian model ACCESS1.0 and the British model HadGEM2-CC project the lowest damages. The models that predict low damages in the early part of the record also predict low damages in the late part of the record; the models that predict high damages early also predict high damages later in the record. Gershunov et al.^18^ identified a subset of five GCMs (labelled in Table S1 as “Real 5”) that most realistically captured historical AR landfalling activity and contributions of ARs to total annual precipitation in the western United States. The statistical damage model applied to these five models projects greater increases in damages over the course of the twenty-first century than the 16-model ensemble: $1.0 billion in the 1990s, $1.9 billion in the 2020s, to $3.6 billion in the 2090s (Fig. S2).

### Damage projections by AR ranking

A ranking scheme developed by Ralph et al.^32^, similar to familiar scales for hurricanes and tornadoes, can be used to classify ARs into five classes based on peak IVT and duration. AR intensity is commonly quantified by IVT which measures the speed and quantity of water vapor moving through the air column above a given location. In absolute terms, an IVT threshold of 250 kg m^−1^ s^−1^ is typically used to indicate AR conditions; IVT over 1000 kg m^−1^ s^−1^ is associated with extreme or exceptional ARs.

**Figure 4:**
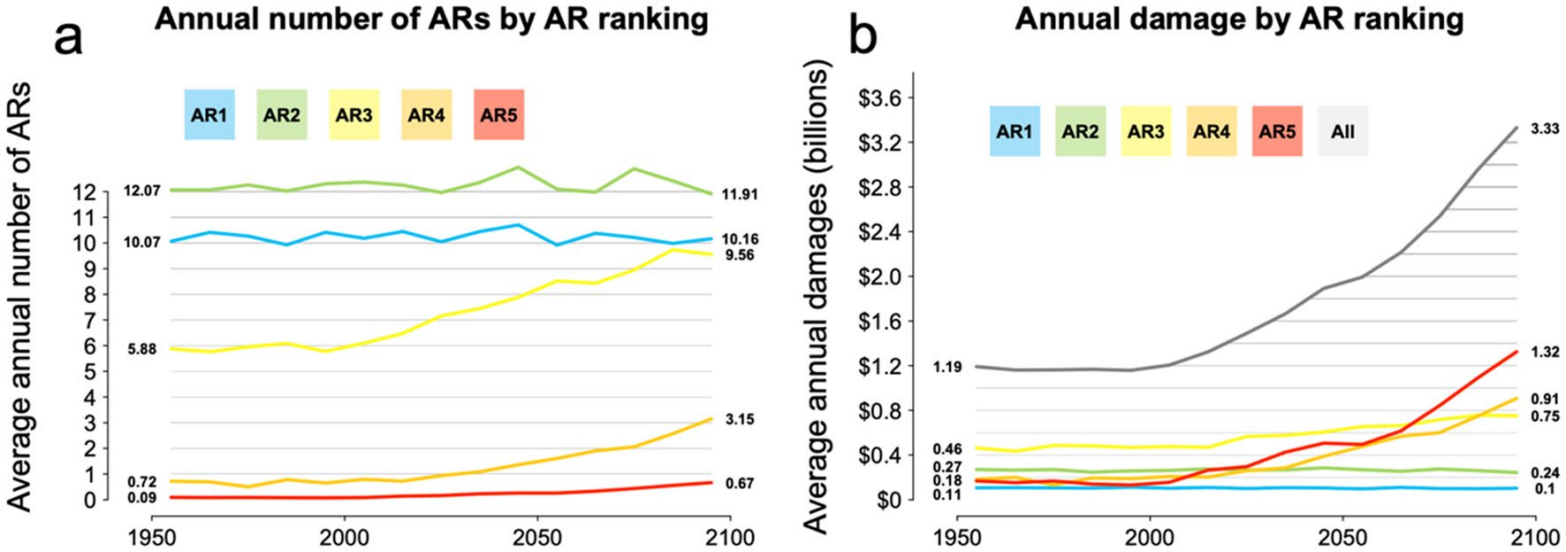
Number of ARs per year by AR scale by decade (**a**). AR1 and AR2 storms remain roughly constant in frequency at 10 and 12 per year respectively from the 1950s to the 2090s. AR3 storms increase in frequency from 6 to 10 per year, AR4 from 0.7 to 3.2, and AR5 from 0.1 to 0.7 per year. Annual damage by AR scale by decade (**b**). AR1 and AR2 storms remain roughly constant in total damage at $100 million and $250 million per year respectively from the 1950s to the 2090s. Damages associated with AR3 storms increase from $460 million to $750 million. Damages associated with AR4 storms increase from $180 million to $910 million. Damages associated with AR5 storms increase from $180 million to $1.32 billion. Total damages increase from $1.2 billion to $3.3 billion per year. Solid lines indicate the mean number of events per year in each decade averaged across 16 different GCMs.

Landfalling ARs in the 16 catalogues were classified locally at coastal grid cells by AR rank. The average annual number of storms in each category was calculated by decade from 1950 to 2100. Weak AR1 and moderate AR2 storms are projected to make landfall over western North America with relatively stable frequency over the 150-year model period: approximately 10 and 12 storms per year, respectively (Fig. 4). Over the 150-year period, strong AR3 storms nearly double in frequency from 6 to 10 storms per year. Extreme AR4 storms quadruple in frequency from 0.7 to 3.2 storms annually. Exceptional AR5 storms increase in frequency from roughly one storm per decade to two storms every three years, a seven-fold increase in frequency. This shift is consequential as flood damages associated with ARs have been found to increase exponentially with AR rank: for each increase in AR rank, median damages increase by roughly a factor of 10, from under $100,000 for AR1 storms to over $250 million for AR5 storms ^8^. The increase in the frequency of extreme AR4 and exceptional AR5 storms simulated in the GCMs drives the nearly threefold increase in expected damages due to ARs over the twenty-first century.

### County-level damage projections

To identify regions at greatest risk of increased AR intensity, location-specific damage models were constructed at the county level across the 11 western conterminous states. Damages were then aggregated by decade across the 16 RCP8.5 GCMs to provide spatially disaggregated projections of future flood impacts across the western United States. Two simple metrics may be used to compare future damages to past or present damages: the difference and the ratio (or percent change). Changes in damage as represented by differences tend to be greatest in those counties with the greatest baseline damages, owing to high populations and property values (e.g., Los Angeles County) or to large numbers of properties exposed to flood risk (e.g., Sonoma County where many properties are situated in the floodplain of the lower Russian River).

**Figure 5:**
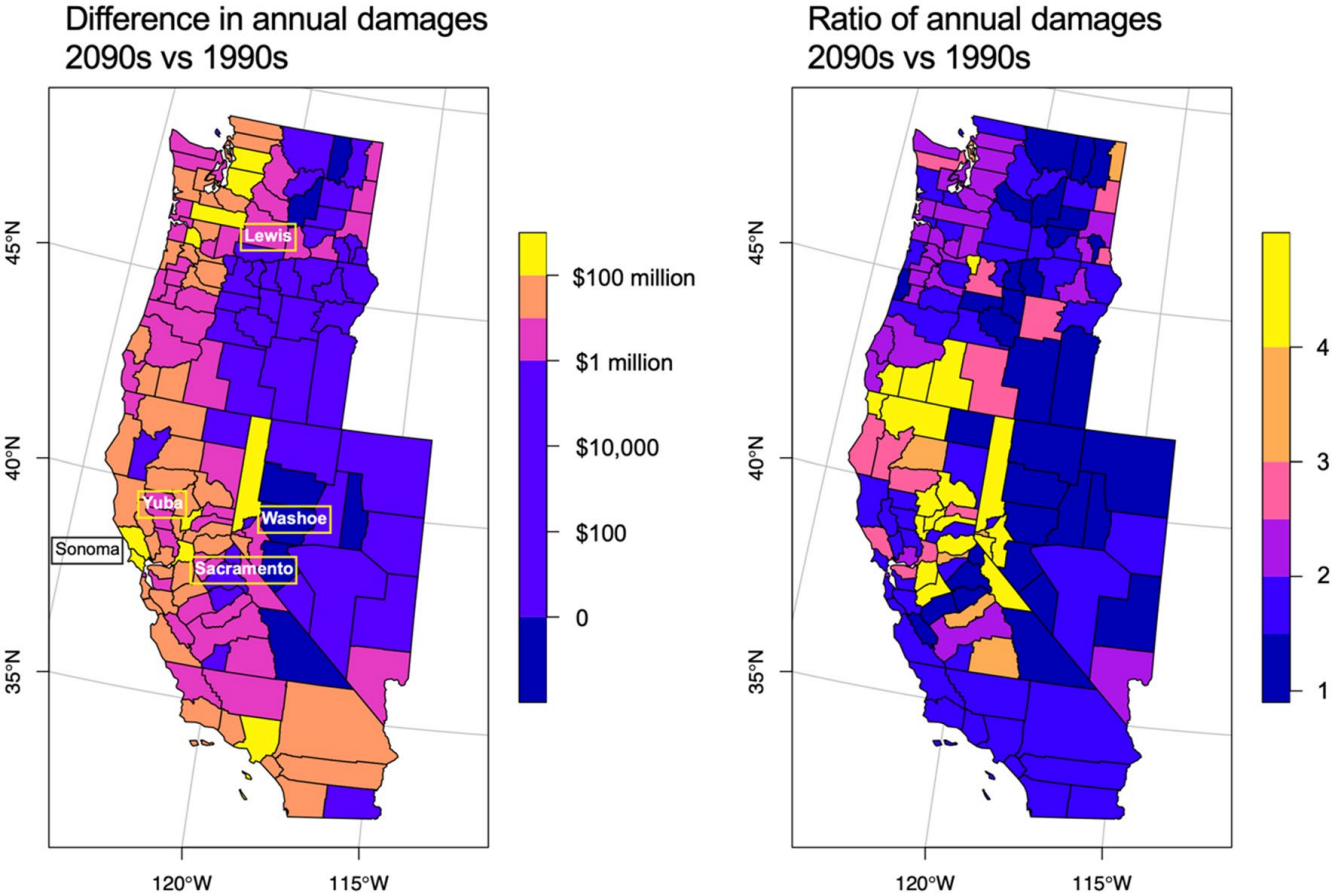
Counties most affected by changes in AR-related flood damages: differences in annual damages by county in the 2090s compared to the 1990s. Flood damages associated with ARs across the western 11 states are projected to be uniformly higher in the 2090s compared to the 1990s. The most significant increases are observed in the coastal regions, the Sierra Nevada range, and in more populated counties. The highest ratios of damages in the 2090s to damages in the 1990s are observed in the Sierra Nevada range and along the border of California and Oregon.

The greatest increases in damage levels are projected to occur in near-coastal regions, the western side of the Sierra Nevada range, and along the Columbia River basin. The counties projected to experience the greatest increases in damages from the 1990s to the 2090s are Sonoma California, Washoe Nevada, Lewis Washington, Sacramento California, and Yuba California (Fig. 5). These are not the most populated counties in the western United States but have development concentrated along rivers prone to significant flooding: the Russian River in Sonoma County, the Truckee in Washoe County, the Chehalis in Lewis County, the Sacramento, the San Joaquin, and the American in Sacramento County, and the Yuba and the Feather in Yuba County (see Tables S3 and S4 for results for the top 20 counties, and Data S1 for full results).

In terms of percent changes in damages, the greatest increases are projected to occur in those counties near the Sierra Nevada range and California's Central Valley, and in counties along the border of California and Oregon, the focal point of strong AR landfalling activity^30^. Damage ratios are useful in that they indicate relative changes in risk over time, but they can be sensitive in counties with low baseline damages in the ratio denominators. Of the 348 counties with non-zero insured flood damages in the historical record, all but one are projected to experience increased flood damages by the 2090s relative to the 1990s (damages in Sanders County, Montana, are projected to decrease by 9% though the decrease is not statistically significant).

## Discussion

There are several sources of uncertainty in AR-related flood damage projections, including climate model uncertainty, climate scenario uncertainty, AR detection technique (ARDT) uncertainty, and damage model uncertainty. Here, climate model uncertainty is explicitly addressed by generating damage projections based on 16 GCMs. Uncertainties associated with different climate scenarios are addressed by considering both the RCP4.5 and RCP8.5 scenarios. Under the 2015 UNFCCC Paris Agreement, 192 countries agreed to limit emissions to keep increases in global temperatures below 2 °C, preferably to 1.5 °C, relative to pre-industrial levels. The 2 °C goal is associated with the RCP2.6 trajectory while the 1.5 °C is associated with a more ambitious RCP1.9 trajectory. As of 2021, the Paris Agreement pledges, even if fully achieved, were expected to fall short of what is required to limit the global temperature rise to 1.5 °C^33^. Hence, it is important to consider the impacts associated with the greater warming scenarios of RCP4.5 and RCP8.5. As the next-generation CMIP6 GCM outputs become available, the analyses developed here could be extended to consider a larger set of models and warming scenarios.

Over twenty ARDTs have been developed^34^. This study uses the Gershunov et al.^30^ SIO-R1 automated ARDT which has been validated against observational precipitation records in the western United States. Furthermore, damage relationships developed for three separate ARDTs^8,24,35^ exhibit a high degree of consistency indicating some robustness of the damage projections to the choice of ARDT. While there are differences in projected climate impacts on ARs based on ARDT, the general predictions are similar^19^.

This study relies on NFIP insured loss data linked to NWS-derived total damage measures to estimate direct flood damages. This method has several limitations including the low penetration rate in the flood insurance market^25^. Other sources of total flood damage data such the Spatial Hazards Events and Losses Database for the United States (SHELDUS^36^) are more comprehensive but not of sufficiently fine temporal resolution to analyse AR impacts. Using SHELDUS data in place of older NWS data to calibrate total damage estimates would generate different absolute damage projections, though the spatial patterns and temporal trends identified here would be similar as the SHELDUS and NWS datasets make use of similar input data.

The damage model presented here aggregates impacts over counties. Damaging flood events range from highly localized to multi-basin events. IVT is a key input variable in the damage model and is resolved at 2.5° (111 km longitude by 73–94 km latitude) which is sufficient to capture daily AR location and intensity^37^. While locally concentrated flood damages can result from extreme precipitation over short durations^38^, large-scale spatial patterns of long-term trends in damages are likely to be well represented by our statistical models. Physical modelling tools could be used to generate flood damage projections by explicitly modelling the hydrologic response and economic impacts of changing atmospheric conditions^39^ though such an approach would require significant data curation efforts and computing resources to implement over the western United States.

Sixteen models over 150 years represent 2400 synthetic years of damages. The insurance records on which the damage model is based extends only 40 years so extreme damage years may not be well captured by this analysis. The ARkStorm analysis^40^ predicted that a 1 in a 1000-year series of ARs would generate flood-related damages of $860 billion in California (2020 USD). No damages of this magnitude were observed in projections. This is a limitation of the modelling strategy that could potentially be overcome with a more fine-grained effort incorporating probabilistic estimates of stream flow and dam and levee failures, or by applying probability models to estimate damages associated with 100-year and 1000-year events.

Here, the impacts of projected changes in AR frequency and intensity on flood damages have been estimated while holding exposure and sea level constant. The focus of this analysis is to quantify the economic impact of increasing AR intensity, boosted by climate change, at the county level. The models do not adjust for projected population changes or the compound effects of increasing AR intensity and sea level rise, both of which may increase damages^41^. The models also do not account for policy responses that harden flood defences and increase community resilience which may reduce future damages by mitigating the increased risk of flooding due to increasing AR intensity.

Our models use GCM projections linked to historic records of flood damages to predict the economic impacts of changes in extreme weather patterns and identify locations at greatest future risk of damage. This allows local and regional planners to target adaptation investments to the areas that will result in the greatest net benefits. As the intensity distribution of severe storms shifts to favour events in the extreme tail of the distribution, overall damages are expected to increase nonlinearly. Our models project that under the RCP8.5 scenario, flood damages due to ARs could approximately triple by the end of the twenty-first century relative to the late twentieth century baseline, holding spatial patterns of exposure and vulnerability constant. Limiting greenhouse gas emissions to achieve the RCP4.5 scenario could reduce end-of-century damages relative to the RCP8.5 scenario by approximately 30%, i.e., approximately resulting in a doubling instead of a tripling of damages. The continued development of observational and modelling capabilities^42,43^ will be critical in directing resources to protect vulnerable communities and to mitigate the social and economic costs of climate-related intensification of ARs in the western United States.

## Supplementary Information

Supplementary Information 1.

Supplementary Information 2.

## Data Availability

All data required to understand and assess the conclusions of this research are available in the supplementary materials and referenced works. All data, code, and materials will be made available to any researcher seeking to reproduce or extend the analysis. All results, figures, and tables were generated using R version 4.0.3 (https://www.r-project.org/).
