# Supplementary material for: Climate change contributions to future atmospheric river flood damages in the western United States
Source: Sci Rep. 2022 Aug 12;12:13747. doi: 10.1038/s41598-022-15474-2 (PMC9374734; doi:10.1038/s41598-022-15474-2)
Supplement: Supplementary file 2 — Supplementary Information 2. [file 41598_2022_15474_MOESM2_ESM.pdf]

# Supplementary Materials for

## Climate change contributions to future atmospheric river flood damages in the western United States

Thomas W. Corringham, James McCarthy, Tamara Shulgina, Alexander Gershunov, Daniel R. Cayan, F. Martin Ralph

Correspondence to: tcorringham@ucsd.edu

**This PDF file includes:**

Materials and Methods
Figures S1–S2
Tables S1–S4

**Other Supplementary Materials for this manuscript include the following:**

**Data S1**

## Materials and Methods

Property loss data

NFIP loss data comprise daily claims from 1978 to 2017, with each claim located to the nearest NFIP community (city, typically, or county remainder) listing the total insured loss to the building and its contents by claim. All monetary values are adjusted for inflation to 2020 U.S. dollars (*44* US FRED). A comparison of 1983 to 2003 annual NFIP losses to an NWS compilation of economic impacts of flooding (*25* Corringham and Cayan 2019; *27* Downton et al., 2005) established that insured losses are a good proxy for overall economic impacts. The NWS data comprise annual total estimated damages due to flooding at the state level from 1983 to 2003, as reported by newspaper articles and the reports of federal agencies.

In the 11 western states, NFIP-insured losses account for roughly 1/30 of western total direct damages as estimated by the NWS; the Pearson correlation between the two time series is 0.8. In this study, the 30-fold difference between insured losses and total impacts was used to provide an estimate of total economic impacts associated with flood events. Given the low spatial and temporal resolution of the NWS data, the total damage estimates are highly imprecise. The measures of insured flood losses, on the other hand, are exact and easily comparable over space and time.

Although the NFIP data have several attractive features, they also suffer from significant limitations. Participation rates are low in the western United States (*45* Dixon et al., 2005), even in relatively high-risk areas, so the numbers of claims and insured losses are imperfect measures of total damages. Several biases are expected. The NFIP program covers only residential property so floods that cause disproportionate damage to agriculture, infrastructure, and industrial plants will be downweighted in this analysis. Older properties receive subsidies and are

likely overrepresented in the portfolio of risks. This is one of many distortions in the NFIP. The market for insurance is not a free market and does not behave like one (*46* Michel-Kerjan et al., 2010). These caveats aside, the NFIP data are highly resolved temporally and spatially specific, so they provide a useful source with which to assess the economic impacts of flood events associated with ARs and extreme hydrologic events more generally.

As of policy year 2017, there were approximately 392,000 policyholders in the 1807 participating NFIP communities in the 11 western states. The total coverage in force was $118 billion. Total premiums paid in 2017 were $292 million, or 0.247% of total coverage in force. The average policyholder paid an annual premium of $745 for $301,000 coverage. Further summary statistics of the NFIP data are given in Corringham and Cayan (2019) (*25*). To analyse the impacts of ARs on insured losses, NFIP loss data and total damage estimates were aggregated spatially by county (Figure 4). Daily claims and insured losses were used throughout, with days defined in local time for the NFIP dataset.

<u>Insured losses normalized by NFIP coverage levels</u>

In the 11 western states, annual NFIP coverage levels varied non-monotonically over the historical model training period (*25* Corringham and Cayan, 2019). Of, concern is that if losses increased because of non-climatic drivers, e.g., increases in exposure, and ARs increased in intensity concurrently, the future effects of changing AR intensity could be overestimated. To account for the possibility that trends in coverage levels were influencing results, a supplementary set of analyses was conducted using West-aggregated NFIP insured loss payments divided by annual NFIP coverage in force over the 11 western states and county-aggregated NFIP insured losses divided by county-aggregated annual NFIP coverage in force. The results were qualitatively similar to the forecasts of unadjusted damages (Figures 1 and S1):

insured losses per $100,000 coverage in the RCP8.5 “high emissions” scenario doubled by the 2050s and more than tripled by the 2090s relative to the baseline period of the 1990s. Insured losses per coverage in the RCP8.5 scenario match RCP4.5 “intermediate emissions” levels closely until mid-century and then diverge, as in the total damages models (Figure 1).

The similarity in results may be due to insured losses tracking the intensity of the hazard more closely than the exposure over the 40-year historical period. Exposure is important, as found in Corringham et al. (2019) (*8*): an AR5 that impacts Southern California causes more damage than an AR5 that impacts Southern Oregon, other things being equal (e.g., antecedent soil moisture). However, in any given county or region, the variability in insured losses over time is better explained by the variability in event intensity than by variability in exposure.

In the observational NFIP data, coverage increased significantly over time in almost all counties. Annual insurance payments were highly variable exhibiting no significant upward or downward trend over the study period. Payments per coverage decreased slightly over time. This could be because of secular decreases in vulnerability or because of the stochastic nature and low frequency of extremely damaging flooding events in the western U.S. (e.g., the 1982/83 and 1997/98 El Niño years were not matched in hazard intensity by the 2015/16 El Niño years). County-specific regression models including county-annual coverage as a predictor did not change county-specific model results. In a panel regression analysis, the cross-sectional correlation in coverage and payments was highly significant but within-county time series correlation was minimal. Hence, changes in coverage, or exposure, are important, but the temporal variability in insured losses in the western U.S. appears to be driven by the temporal variability in the hazard, predominantly ARs, rather than by the temporal variability in coverage levels.

AR data

A set of 16 hindcast and forecast AR catalogues were obtained from Gershunov et al. (2019) (*18*). Table S1 provides information on each model: name, origin, and spatial resolution. An automated AR detection scheme (*30* Gershunov et al., 2017) was applied on a 6-hourly timescale to an ensemble of 16 global climate models (GCMs, Table S1) over the historical period (1950– 2005) and future (2006–2100) projected under Representative Concentration Pathway 8.5 (RCP8.5), the "high emissions" scenario, from Phase 5 of the Coupled Model Intercomparison Project (CMIP5) (*31* Taylor et al., 2012). Gershunov et al. 2019 (*18*) used the 16 GCMs for which data were available for computing the vertically integrated horizontal vapor transport (IVT) – a key variable for defining and detecting atmospheric rivers (ARs).

The daily simulations of historical (1950–2005) and projected (2006–2100) specific humidity, zonal and meridional wind components at four standard pressure levels spanning 1000–500 millibars (1000, 850, 700 and 500 mb) were used to estimate IVT and detect ARs making landfall along the west coast of North America (20–60°N). Landfalling ARs were detected at the native resolution of each GCM, and the catalogues were interpolated spatially to a common grid (2.5° × 2.5°) and temporally to a standard calendar.

The methodology to detect ARs is that of Gershunov et al. (2017) (*30*, SIO-R1), which has been applied to the National Center for Environmental Prediction and National Center for Atmospheric Research (NCEP/NCAR) reanalysis (*47* Kalnay et al., 1996) and validated with independently observed precipitation data (Gershunov et al. 2017). The SIO-R1 catalogue is recognized for its relevance for west coast precipitation studies, and it compares favourably with other available AR catalogues for these applications (*34* Shields et al., 2018; *37* Ralph et al., 2018). Specifically, this 2.5° × 2.5° catalogue agrees well with those based on more finely

resolved reanalyses and provides a longer historical record for GCM validation (1948-present) than most other reanalysis products.

Methods

Insurance claims and damages were aggregated daily over the western 11 conterminous states, and individually over 414 counties. The SIO-R1 catalogue of observed ARs was matched to daily damages over the West, by county and by region from January 1978 to December 2017. Latitude was binned into 2.5° bands from 22.5°N to 57.5°N. Total IVT was calculated at coastal grid cells at each latitude over the course of the event for each AR, summed over 6-hour intervals. Claims and damages were modelled as functions of landfalling total IVT at each latitude, indicator variables for 1 to 5 AR ranking, and indicators for month of year or season, and a variable indicating the number of days since the last atmospheric river of rank 3 or above, binned into categories of 1 day, 2-4 days, and greater than 5 days, to capture the effects of antecedent hydrologic conditions. Other specifications were considered, but this specification achieved a balance between in-sample model fit and parsimony. For each GCM, damages were then predicted for each AR based on landfalling total IVT at each latitude, indicator variables for AR category and month of year, and the time since last AR. This yielded a set of 16 hindcast and forecast AR damage time series from 1950 to 2100. AR damages were then aggregated annually by GCM yielding a set of 16 annual AR damage time series.

The historical AR data cover the period of 1948 to 2018 at a 6-hourly timestep. Each entry is a location-timestep associated with an AR which is given a unique AR identification number. Each of the 16 projections is a catalogue of ARs detected from 16 CMIP5 GCMs using the Gershunov et al. (2017) (*30*) detection methodology. Each entry is numbered by a unique AR identification number and provides 6-hourly meridional and zonal components of IVT at the

location of landfall. Each set of projections from 2006 to 2100 (2006 to 2099 in three models) was combined with its associated set of hindcasts from 1950 to 2005, yielding 151 (or 150) years of data for each of 16 GCMs.

To make the historical data comparable to the projections and hindcasts, the historical data were aggregated to daily timestep, taking the maximum IVT per AR-day as the representative value. Other specifications using mean IVT or IVT at hour zero yielded datasets with too few high category storms using the Ralph et al. 2019 (*32*) AR scale relative to expectations and were not included in the analysis. In both the hindcast and projection data sets, latitudes were limited to 27.5°N to 47.5°N. If an AR passed into this range, the entire AR was retained, resulting in IVT observations from 22.5°N to 57.5°N.

All data sets were aggregated using the same procedure in which each AR was listed as a single observation comprised of the unique AR identification number, the start and end dates, the duration in hours (all multiples of 24, as everything was aggregated daily), the starting, ending, minimum, maximum, and mean landfalling latitude, the mean and maximum daily IVT over the course of the event, the AR ranking from 1 to 5, and the storm total IVT at each latitude band from 22.5°N to 57.5°N. This allowed for the construction of models with up to 25 basic features, and additional derived features. The historical data were then matched to NFIP claims and insured losses, and total damages.

Linear ordinary least squares regression models were constructed using the historical data, linking storm total IVT at each latitude, 1-to-5 AR rank, a categorical representation of the number of days since the last AR3, AR4, or AR5 event, and a set of month-of-year or season indicator variables. Machine learning algorithms were also applied to the data but failed to produce statistically significant improvements in cross-validated out-of-sample model fit over

the linear model. A model that predicted the hyperbolic sine of damages (like the logarithm, but well-defined for input values of zero) as a linear function of the inputs yielded a higher coefficient of determination, unsurprisingly given the skew of the damage distribution, but generated unrealistic damage projections and so was not used. Models that fitted a sigmoid response function mitigated this issue, but were highly sensitive to the maximum damage threshold, for which a good estimate is not available.

Hence, the simple linear ordinary least squares damage model was selected. The coefficient of determination on a West-aggregated ordinary least squares linear model was 0.1954 indicating that the simple model captured 20% of the variance in total damages (Table S2). This is in line with other insured flood damage models at this level of spatial resolution (*48* Corringham, 2018). In addition to West-aggregated models, several spatially disaggregated models were constructed. The preferred model predicted county damage aggregates as independent functions of storm-total IVT values at each latitude, AR rank, days since last AR3 or greater, and a month-of-year effect.

Using the estimated model parameters, damages were estimated for each AR in each of the 16 synthetic catalogues derived from the GCMs. To compare damages over time, each of the 16 AR damage time series was aggregated annually over the West and by county yielding 16 annual time series of damages by geography. Damages were compared by grouping the annual time series into moving windows of decades centred around the year of interest. In this way empirical distributions of 160 observations (16 models times 10 years) were created.

Nonparametric Kolmogorov Smirnov tests of the equality of distributions were applied to the empirical distributions by decade. Percent changes in damages were calculated relative to the 1990s by decade from the 2050s to 2090s. A similar annual aggregation procedure was applied

to the county aggregates. In this case, as with the West-aggregated case, we compared the decade of 1990-2000 to the decade of 2090-2100 to consider the maximum effect of changes in modelled AR intensity over the 150-year period (Table 1). For decision makers, flood managers, public agencies, real estate and sovereign wealth investors, and regulators, shorter time horizons are more useful. For these decision makers we generated forecasts of damages over the next 30 years at a 5-year time step (Table 2).

The damage models and projections reflect only changes in the frequency and intensity of atmospheric rivers, abstracting away from changes in exposure and sea level rise. Future research is needed to explore the impacts of exposure, policy changes, and the interactions between sea level rise and increasingly intense atmospheric rivers.

**Supplementary References**

44. U.S. Federal Reserve Bank of St. Louis. *Personal Consumption Expenditures (implicit price deflator) DPCERD3Q086SBEA* (FRED, 2020).

45. Dixon, L., Clancy, N., Seabury, S. A. & Overton, A. *The National Flood Insurance Program's Market Penetration Rate Estimates and Policy Implications* (RAND Corporation, 2006).

46. Michel-Kerjan, E. O. et al. Catastrophe economics: The national flood insurance program. *J. Econ. Perspect.* **24**, 165–186 (2010).

47. Kalnay, E. et al. The NCEP/NCAR 40-year reanalysis project. *Bull. Am. Meteor. Soc.* **77**, 437–470 (1996).

48. Corringham, T. W. *Wildfires, Floods, and Climate Variability* (University of California, San Diego, 2018).

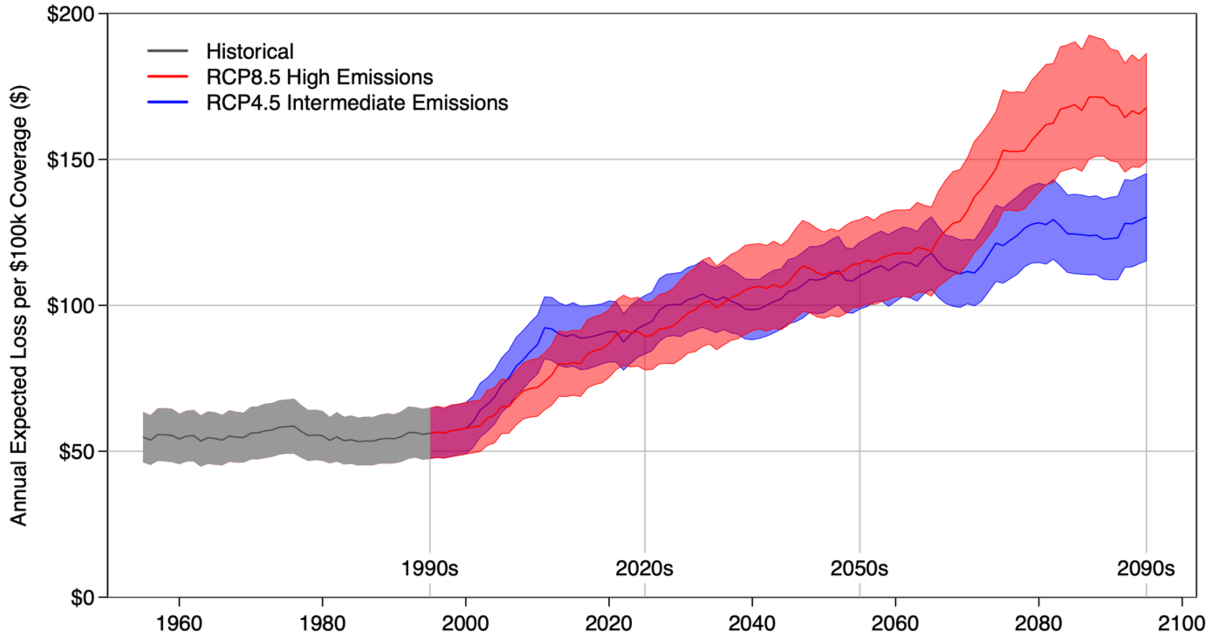


**Figure S1.**

Normalized projections of AR-related flood insured losses per West-aggregated $100,000 of NFIP coverage are generated using a loss model linked to 14 GCMs for which sufficient RCP4.5 and RCP8.5 data were available. Expected annual loss per coverage values remain stable at approximately $56 over the historical period of 1950 to 2005 and then rise to $90 by 2020 and $110 by 2050. Beyond 2050, normalized damages associated with the RCP4.5 "intermediate emissions" and RCP8.5 "high emissions" scenarios begin to diverge. By the 2090s, insured losses per $100,000 coverage are projected at $130 and $168 for the RCP4.5 and RCP8.5 models, respectively. Means and 95% standard errors are taken over centred 10-year moving windows of observations from the 14-model ensemble.

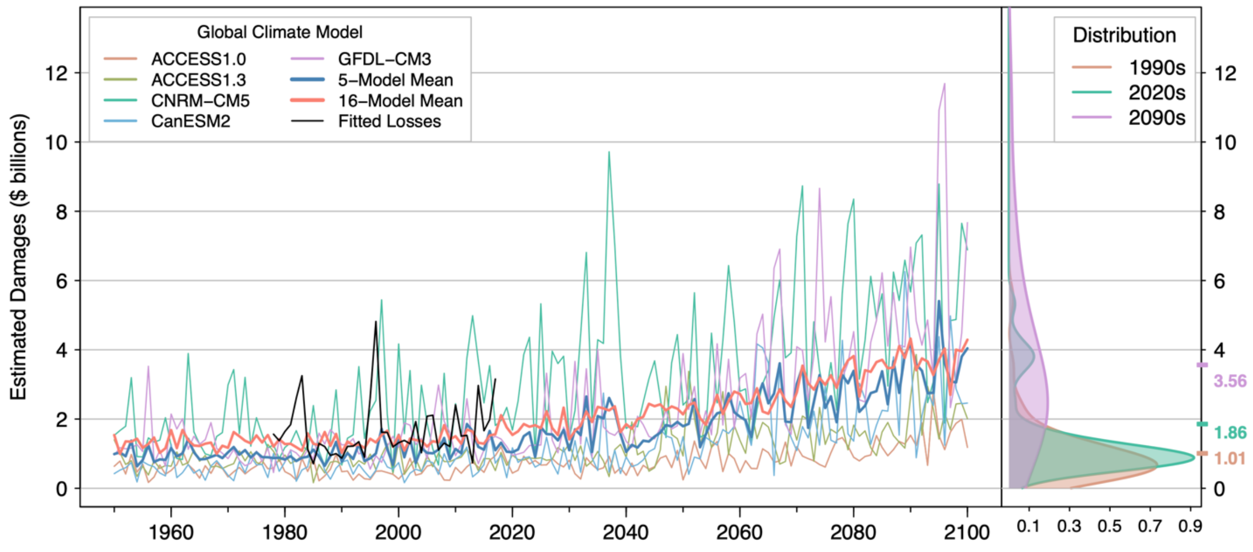


**Figure S2.**

The "Real 5" RCP8.5 climate models (see Table S1) combined with the damage model based on the observational record project an increase in mean annual damages over the 11 western states from $1.01 billion in the 1990s to $1.86 billion in the 2020s to $3.56 billion in the 2090s. CNRM-CM5 and GFDL-CM3 predict higher annual damages. ACCESS1.0 and ACCESS1.3 predict lower annual damages. CNRM-CM5 predicts damages in line with the 5-model and 16-model mean predictions. Gaussian kernel densities of damages by decade across the 5 models reveal increasing damages over time. Kolmogorov-Smirnov tests on the differences in annual damage distributions in the 2020s and 2090s relative to the 1990s yield p-values of 0.068 and $5 \times 10^{-11}$, respectively.

**Table S1.** Global climate models. 16 GCMs were used in the full RCP8.5 analyses. 14 of these were used in the RCP4.5 versus RCP8.5 comparisons (Figure 1 and Figure S1). RCP4.5 data for GFDL-CM3 and MIROC5 were not available at the time of analysis due to technical reasons. Five models (Real 5) that most realistically captured historical AR landfalling activity and contributions of ARs to total annual precipitation in the western United States are indicated in the table, as are the spatial resolutions of the GCMs, all of which were resampled to provide 2.5-degree gridded IVT for damage models (*18* Gershunov et al., 2019; *30* Gershunov et al., 2017).

| # | Model | Institution | Latitude resolution | Longitude resolution |
|---|---|---|---|---|
| 1 | *ACCESS1.0* ***(Real 5)*** | *CSIRO (Commonwealth Scientific and Industrial Research Organization), and BOM (Bureau of Meteorology), Australia* | *1.25°* | *~1.88°* |
| 2 | *ACCESS1.3* ***(Real 5)*** | *CSIRO and BOM, Australia* | *1.25°* | *~1.88°* |
| 3 | BCC-CSM1.1 | Beijing Climate Center, China Meteorological Administration, China | ~2.79° | ~2.81° |
| 4 | *CanESM2* ***(Real 5)*** | *Canadian Centre for Climate Modelling and Analysis, Canada* | *~2.79°* | *~2.81°* |
| 5 | *CNRM-CM5* ***(Real 5)*** | *Centre National de Recherches Météorologiques / Centre Européen de Recherche et Formation Avancées en Calcul Scientifique, France* | *~1.4°* | *~1.41°* |
| 6 | *GFDL-CM3 ** ***(Real 5)*** | *Geophysical Fluid Dynamics Laboratory, United States* | *2°* | *2.5°* |
| 7 | GFDL-ESM2G | Geophysical Fluid Dynamics Laboratory, United States | ~2.02° | 2.5° |
| 8 | GFDL-ESM2M (Missing 1995- 2005) | Geophysical Fluid Dynamics Laboratory, United States | ~2.02° | 2.5° |
| 9 | HadGEM2-CC | Met Office Hadley Centre, United Kingdom | 1.25° | ~1.88° |
| 10 | Inmcm4 | Institute for Numerical Mathematics, Russia | 1.5° | 2° |
| 11 | IPSL-CM5A-LR | Institut Pierre-Simon Laplace, France | ~1.89° | 3.75° |
| 12 | IPSL-CM5A- MR | Institut Pierre-Simon Laplace, France | ~1.27° | 2.5° |
| 13 | MIROC5 * | Atmosphere and Ocean Research Institute (The University of Tokyo), National Institute for Environmental Studies, and Japan Agency for Marine-Earth Science and Technology, Japan | ~1.4° | ~1.41° |
| 14 | MIROC-ESM | Japan Agency for Marine-Earth Science and Technology, Atmosphere and Ocean Research Institute (The University of Tokyo), and National Institute for Environmental Studies, Japan | ~2.79° | ~2.81° |
| 15 | MIROC-ESM-CHEM | Japan Agency for Marine-Earth Science and Technology, Atmosphere and Ocean Research Institute (The University of Tokyo), and National Institute for Environmental Studies, Japan | ~2.79° | ~2.81° |
| 16 | MRI-CGCM3 | Meteorological Research Institute, Japan | ~1.12° | ~1.13° |

* Models not available for RCP4.5

**Table S2.** Regression table, observational record, damage per AR as the dependent variable.

| | Estimate | Standard Error | *t* Statistic | *p* Value |
|---|---|---|---|---|
| Intercept | 74000 | 806000 | 0.092 | 0.9267 |
| February | 405000 | 757000 | 0.534 | 0.5931 |
| March | -617000 | 790000 | -0.781 | 0.4349 |
| April | -1170000 | 843000 | -1.39 | 0.1635 |
| May | -1080000 | 882000 | -1.23 | 0.2195 |
| June | -632000 | 915000 | -0.69 | 0.4903 |
| July | -273000 | 1050000 | -0.26 | 0.795 |
| August | -221000 | 1040000 | -0.212 | 0.8317 |
| September | -1200000 | 836000 | -1.44 | 0.1505 |
| October | -1400000 | 741000 | -1.88 | 0.06 |
| November | -937000 | 715000 | -1.31 | 0.1903 |
| December | 42700 | 715000 | 0.0597 | 0.9524 |
| AR2 | -867000 | 702000 | -1.24 | 0.2167 |
| AR3 | -397000 | 854000 | -0.466 | 0.6417 |
| AR4 | 4190000 | 1650000 | 2.53 | 0.0115 |
| AR5 | 58600000 | 5420000 | 10.8 | $< 10^{-8}$ |
| 2–4 days since AR | 3350000 | 1020000 | 0.338 | 0.736 |
| 5+ days since AR3 | 52000 | 930000 | 0.056 | 0.955 |
| Storm total IVT 22.5 N | -11100 | 2320 | -4.76 | $2.13 \times 10^{-6}$ |
| Storm total IVT 25 N | -685 | 1120 | -0.61 | 0.5418 |
| Storm total IVT 27.5 N | 1980 | 771 | 2.56 | 0.0105 |
| Storm total IVT 30 N | 1180 | 980 | 1.21 | 0.228 |
| Storm total IVT 32.5 N | 6120 | 1120 | 5.48 | $5 \times 10^{-6}$ |
| Storm total IVT 35 N | 2500 | 960 | 2.6 | 0.0094 |
| Storm total IVT 37.5 N | 3530 | 651 | 5.43 | $7 \times 10^{-6}$ |
| Storm total IVT 40 N | 791 | 700 | 1.13 | 0.2587 |
| Storm total IVT 42.5 N | 952 | 676 | 1.41 | 0.1597 |
| Storm total IVT 45 N | 2640 | 602 | 4.38 | $1.3 \times 10^{-5}$ |
| Storm total IVT 47.5 N | 1600 | 826 | 1.93 | 0.0534 |
| Storm total IVT 50 N | -103 | 729 | -0.141 | 0.888 |
| Storm total IVT 52.5 N | -727 | 882 | -0.824 | 0.41 |
| Storm total IVT 55 N | 1040 | 1250 | 0.83 | 0.4068 |
| Storm total IVT 57.5 N | -249 | 1340 | -0.186 | 0.8521 |

All numbers to 3 significant digits. $F$-statistic: 11.5 on 30 and 1421 degrees of freedom, $p$-value $< 10^{-16}$

**Table S3.**

Changes in insured losses and estimated damages due to flooding associated with atmospheric rivers from 2020 to 2050.

| Rank | County | State | Difference in Damages 2020 to 2050 (Millions $) | Difference in Insured Losses 2020 to 2050 (Thousands $) | Ratio of Damages 2020 to 2050 |
|---|---|---|---|---|---|
| 1 | Sonoma | California | 60.3 | 2009.2 | 1.26 |
| 2 | Washoe | Nevada | 58.3 | 1944.2 | 1.50 |
| 3 | Lewis | Washington | 50.8 | 1693.8 | 1.29 |
| 4 | Sacramento | California | 23.4 | 780.1 | 1.29 |
| 5 | King | Washington | 21.6 | 720.1 | 1.28 |
| 6 | Yuba | California | 21.4 | 714.8 | 1.53 |
| 7 | Marin | California | 19.0 | 634.9 | 1.22 |
| 8 | Snohomish | Washington | 17.6 | 586.3 | 1.33 |
| 9 | Columbia | Oregon | 17.5 | 582.2 | 1.33 |
| 10 | Los Angeles | California | 17.3 | 576.9 | 1.18 |
| 11 | Pierce | Washington | 13.3 | 443.6 | 1.34 |
| 12 | Clackamas | Oregon | 11.7 | 390.0 | 1.28 |
| 13 | Napa | California | 11.0 | 366.6 | 1.21 |
| 14 | Stanislaus | California | 9.0 | 299.9 | 1.49 |
| 15 | Douglas | Nevada | 8.6 | 285.9 | 1.52 |
| 16 | Tillamook | Oregon | 8.4 | 280.7 | 1.27 |
| 17 | Skagit | Washington | 8.2 | 273.2 | 1.27 |
| 18 | Placer | California | 8.1 | 271.2 | 1.16 |
| 19 | Sutter | California | 6.8 | 227.5 | 1.52 |
| 20 | Monterey | California | 6.8 | 226.7 | 1.12 |

**Table S4.**

Changes in insured losses and estimated damages due to flooding associated with atmospheric rivers from the 1990s to the 2090s.

| Rank | County | State | Difference in Damages 1990s to 2090s (Millions $) | Difference in Insured Losses 1990s to 2090s (Thousands $) | Ratio of Damages 1990s to 2090s |
|---|---|---|---|---|---|
| 1 | Sonoma | California | 283.2 | 9440.2 | 2.53 |
| 2 | Washoe | Nevada | 275.5 | 9184.0 | 4.47 |
| 3 | Lewis | Washington | 168.7 | 5624.3 | 2.05 |
| 4 | Sacramento | California | 111.3 | 3711.6 | 2.80 |
| 5 | Yuba | California | 103.7 | 3456.3 | 4.89 |
| 6 | Los Angeles | California | 71.5 | 2382.9 | 1.91 |
| 7 | King | Washington | 70.7 | 2355.0 | 2.02 |
| 8 | Marin | California | 63.6 | 2118.6 | 1.85 |
| 9 | Columbia | Oregon | 59.4 | 1981.2 | 2.26 |
| 10 | Snohomish | Washington | 58.7 | 1958.2 | 2.26 |
| 11 | Pierce | Washington | 49.7 | 1656.9 | 2.47 |
| 12 | Stanislaus | California | 43.8 | 1460.6 | 4.55 |
| 13 | Douglas | Nevada | 41.3 | 1377.7 | 4.81 |
| 14 | Napa | California | 40.5 | 1350.4 | 1.95 |
| 15 | Placer | California | 37.7 | 1258.0 | 1.91 |
| 16 | Sutter | California | 33.1 | 1103.6 | 4.80 |
| 17 | Clackamas | Oregon | 30.2 | 1005.6 | 1.76 |
| 18 | Skagit | Washington | 28.8 | 961.5 | 2.09 |
| 19 | Monterey | California | 27.3 | 908.8 | 1.58 |
| 20 | San Joaquin | California | 27.2 | 908.2 | 4.02 |
